# Risk of COVID‐19 infection among mobile extracorporeal membrane oxygenation team

**DOI:** 10.1002/hsr2.981

**Published:** 2022-12-08

**Authors:** Rabee Tawel, Lubna Altawil, Sunil Hassan Koya, Hani Jaouni, Guillaume Alinier, Abdulqadir J. Nashwan, Ahmed Labib

**Affiliations:** ^1^ Medical Intensive Care Unit Hamad General Hospital Doha Qatar; ^2^ Ambulance Service Hamad Medical Corporation Doha Qatar; ^3^ Weill Cornell Medicine‐Qatar Doha Qatar; ^4^ University of Hertfordshire Hatfield UK; ^5^ Northumbria University Newcastle upon Tyne UK; ^6^ Hazm Mebaireek General Hospital Hamad General Hospital Doha Qatar

**Keywords:** coronavirus disease‐2019, extracorporeal membrane oxygenation, healthcare workers, mobile ECMO, transmission, transport

## Abstract

**Background and Aim:**

The transport of coronavirus‐2019 (COVID‐19) patients on extracorporeal membrane oxygenation (ECMO) is a challenging situation, especially for healthcare workers (HCWs), due to the risk of cross‐infection. Hence, certain precautions are needed for their safety. The study aims to evaluate the risk of COVID‐19 transmission to HCWs who transport COVID‐19 patients on ECMO device.

**Methods:**

A retrospective review of adult patients with COVID‐19 infection supported with ECMO and transported by ground route to the Medical Intensive Care Unit (MICU) at Hamad General Hospital (HGH) and a survey of HCWs involved in those cases.

**Results:**

A total of 63 HCWs of the mobile ECMO team were exposed to COVID‐19‐positive patients on 199 occasions. HCWs exposure time was nearly 110 h, and the total transport distance was 1018 km. During the study period, only two of the mobile ECMO HCWs tested positive for COVID‐19. There was zero incidence of transfer‐associated injuries or accidents to HCWs.

**Conclusions:**

The risk of COVID‐19 cross‐infection to the mobile ECMO team seems to be very low, provided that strict infection prevention and control measures are applied.

## 1 BACKGROUND

The novel severe acute respiratory syndrome‐coronavirus‐2 (SARS‐CoV‐2) infection has caused the most significant pandemic in modern medical history. As of September 2022, over 600 million confirmed SARS‐CoV‐2 infections leading to coronavirus disease 2019 (COVID‐19) had been reported, with over 6.46 million related deaths.^1^ However, the accuracy of reported COVID‐19 mortality in some regions has been questioned.^2^

The SARS‐CoV‐2 transmission and significant risk to healthcare workers (HCWs) caring for patients with COVID‐19 have been reported worldwide.^3^, ^4^, ^5^, ^6^, ^7^, ^8^ This risk is particularly higher for frontline HCWs.^4^, ^7^ SARS‐CoV‐2 is transmissible by contact and respiratory droplets. Airborne transmission is possible during aerosol‐generating procedures (AGP).^8^ Several guidelines for the safe handling of COVID‐19 patients have been published.^9^, ^10^, ^11^

Whilst protecting healthcare professionals performing their duty is extremely important, solutions also need to be found to maximize the chances of survival of critically ill COVID‐19 patients. Extracorporeal membrane oxygenation (ECMO) has been suggested for severe acute respiratory distress syndrome (ARDS) secondary to SARS‐CoV‐2 infection.^12^, ^13^ Typically, ECMO is provided in tertiary or university‐affiliated hospitals, and referrals are received within a geographical region or sometimes from abroad. Upon acceptance, the ECMO team will mobilize for bedside assessment and initiation of ECMO at the referring facility. Patients supported on ECMO will be transported to the ECMO center accompanied by the ECMO team.^13^

Dissemination of infection to transport team members and staff from other facilities is a major concern. Direct and close patient contact, mechanical ventilation, airway suctioning, accidental disconnection of the breathing system, or extubation are all potential AGP and may be encountered during transport.^14^, ^15^ In addition, the confined transport environment may increase the risk of contamination.

Transport and retrieval of COVID‐19 patients, particularly those critically ill who are put on ECMO, is challenging. The risk of HCWs cross‐infection may be considerable; hence, specific guidance for HCWs' safety during transfer has been issued.^16^, ^17^, ^18^

The mobile ECMO service at Hamad General Hospital (HGH) (a member of Hamad Medical Corporation (HMC); the premier healthcare provider in Qatar) was established in 2014 in preparation for the Middle East Respiratory Syndrome‐Coronavirus (MERS‐CoV) outbreak.^19^ Before the COVID‐19 pandemic, the ECMO transport team comprised two ECMO consultants, a perfusionist, two ECMO specialist nurses (a scrub plus circulating nurse), and a respiratory therapist. In addition, the emergency medical service (EMS) provided a critical care paramedic (CCP) and two ambulance paramedics. A rapid response vehicle and a designated ECMO ambulance were deployed for each ECMO activation to a referring facility, which was normally an isolation facility from the same governmental healthcare system. During the pandemic, the mobile ECMO team membership was reduced due to the feared high risk of HCWs cross‐infection and overwhelming clinical demands. As a result, one ECMO consultant was deemed enough, and the respiratory therapist was less often included as they were in high demand in the COVID‐19 units.

**Figure 1:**
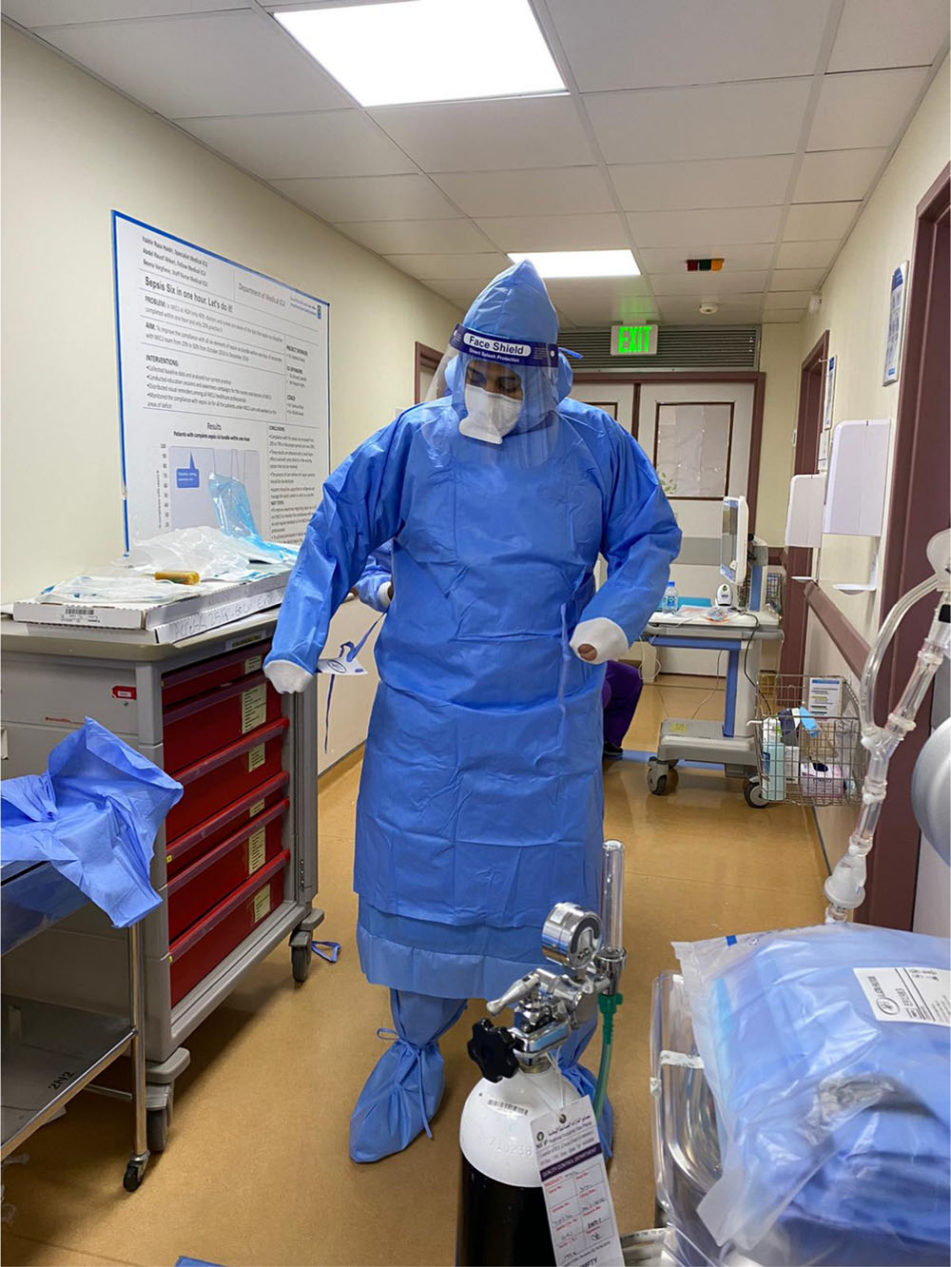
Donning of personal protective equipment outside the cannulation area

**Figure 2:**
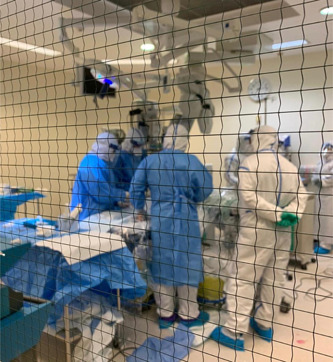
Extracorporeal membrane oxygenation (ECMO) retrieval team performing patient cannulation in a referring facility

The mobile ECMO activation and mobilization is following the local and international guidance.^12^, ^13^, ^20^ Our center encouraged early referral and transport of patients with ARDS.^21^ Our protocol mandated full personal protective equipment (PPE) before entering the COVID‐19 isolation facility (Figure 1). PPE included water‐resistant overhaul, shoe cover, gloves, N‐95 respirator, goggles (or face shield), and headcover. Upon arrival, the team performed a full bedside assessment followed by a multidisciplinary discussion to support an informed decision of either rejection of ECMO, optimization, or cannulation and transfer to the ECMO center at HGH (Figure 2).

When possible, all cannulations were percutaneous, ultrasound‐guided, at the bedside or in the operating theatre with fluoroscopic guidance. After stabilization, patients were transferred with ECMO for pan‐CT scan at the referring facility, to the ambulance, and finally, to the Medical Intensive Care Unit (MICU) at HGH.

We hypothesized that frequent, repeated, and prolonged exposures to COVID‐19 increase the risk of HCWs co‐transmission; thus, this retrospective study investigates the transmission of the novel SARS‐CoV‐2 to HCWs undertaking transport and retrieval on ECMO of COVID‐19 patients. The current study aims to investigate the incidence rate of COVID‐19 among mobile ECMO team members in the State of Qatar over a 10‐month period.

## 2 METHODS

### 2.1 Study design

This retrospective cohort study was approved by the institutional review board (MRC‐01‐21‐029). The STROBE checklist was followed to guide the reporting of the study findings (Supporting Information 1).

### 2.2 Setting

Unidentifiable patient data were collected and analyzed, and the need for patient consent was waived.

### 2.3 Participants

The subjects of the study were patients and HCWs. We conducted a retrospective review of all adult patients with confirmed COVID‐19 supported with ECMO and transported by ground route to the MICU of HGH, Doha, Qatar, between March 1st 2020 and January 1st 2021. No sample size was calucated, we relied on the number of patients seed during the previously mentioned period.

We included all adult patients with confirmed COVID‐19 infection who were cannulated and then transported by ambulance on ECMO to our tertiary center. We did not include patients with non‐COVID pathology or following E‐CPR. All members of the mobile ECMO team who went on any of the call‐outs for assessment, cannulation, or transportation of those patients were invited to participate in the study. To investigate HCWs' infection with COVID‐19, we distributed a consent form and study information sheet to all identified mobile ECMO team members. Upon consent, an electronic link to the survey was sent to all participants.

We report patients' demographics, the severity of their illness, and mobile ECMO team variables. The data extracted for each mission over the duration of the study included patient's demographics, relevant medical history, team composition, and location of referring facility, all of which were retrospectively extracted from the mission reports. The duration of mobile ECMO team members' exposure is defined as the time from entering the referring facility to the time of ECMO plug‐in at the MICU at HGH. Outcome variables are confirmed mobile ECMO team member infection with COVID‐19 during the study period and any untoward transport incidents.

### 2.4 Variables

Continuous variables are expressed as mean/median and standard deviation (SD). Categorical variables are expressed as numbers and proportions.

### 2.5 Statistical methods

Statistical analysis was conducted using Excel version 16.45 (Microsoft Corporation).

## 3 RESULTS

During the study period, 34 mobile ECMO activations and cannulations were performed for patients with confirmed COVID‐19 infection. Patients were predominantly males (30/34) and of relatively young age (mean 47.9, SD 9.25 years). Patients were critically ill with high acute physiology and chronic health evaluation‐II (APACHE‐ II) score (mean 25.9, SD 4.95) and sequential organ failure assessment (SOFA) score (11.5 mean, SD 1.7). Thirty‐three successful veno‐venous and one veno‐arterial ECMO cannulation were accomplished. Patients' demographics, the severity of illness, and clinical characteristics are presented in Table 1.

**Table 1:** Clinical characteristics of the 34 COVID‐19‐positive patients cannulated and transported by the mobile ECMO team

Patients (*N* = 34) variables	Mean (SD) or *n* (%)
Age in years	47.88 (9.25)
Male gender	30 (88.2%)
Female gender	4 (11.8%)
ECMO configuration	
VV‐ECMO fem‐fem	21 (62%)
VV‐ECMO fem‐jug	11 (32%)
VV‐ECMO dual cannula	1 (3%)
VA‐ECMO fem‐fem	1 (3%)
SOFA score	11.5 (1.7)
APACHE‐II score	25.9 (4.95)

Abbreviations: APACHE‐II, acute physiology and chronic health evaluation‐II; fem‐fem, femoro‐femoral; fem‐jug, femoro‐jugular; SOFA, sequential organ failure assessment; VA‐ECMO, veno‐arterial extracorporeal membrane oxygenation; VV‐ECMO, veno‐venous extracorporeal membrane oxygenation.

A total of 63 different mobile ECMO team members were exposed to COVID‐19 patients in a total of 199 episodes of treatment. All of them consented to receiving and completing the online survey for the study. The frequency of mobile ECMO team exposure to patients with COVID‐19 encountered during transport and retrieval missions is summarized in Table 2. Total transport distance and duration is 1018 km and 6593 min (110 h), respectively. Transportation distance and duration are summarized in Table 3. Mobile ECMO team members' variables are presented in Table 4.

**Table 2:** Mobile ECMO medical personnel exposure to COVID‐19 patients by role

Member exposure Member role	Medical personnel exposed (*n*)	Total number of exposures over the study period
ECMO consultant	3	34
Assisting physician	7	9
ECMO nurse	21	68
Perfusionist	7	34
CCP	11	34
Respiratory therapist	14	20
Total	63	199

*Note*: PS: Mobile ECMO team composition: one ECMO consultant, two ECMO nurses, one perfusionist and one CCP/mission. A respiratory therapist was included in 20 missions only. Abbreviations: CCP, critical care paramedic; ECMO, extracorporeal membrane oxygenation.

**Table 3:** Distance from the referring facility to the extracorporeal membrane oxygenation (ECMO) centre at Hamad General Hospital and duration of exposure of the mobile ECMO team members to the COVID‐19 patients

ECMO centre (MICU, HGH) Referring (Isolation) facility	Distance^a^ (km)	Time‐range (min)	Mean duration of mission (min)
Hazem Mebaireek General Hospital (n = 29)^b^	22	124–355	204
The Cuban Hospital (*n* = 5)^b^	76	210–310	272.5

^a^ Distance calculated using Google maps.

^b^ Data of mission duration is missing for one transfer from the Cuban hospital and for two transfers.

**Table 4:** Survey results of the demographic and clinical characteristics of the mobile ECMO team members

Mobile ECMO team variables (*N* = 63)	Median (range) or *n* (%)
Age (year)	38 (24–58)
Male gender	33/58 (56.9%) (5 did not respond)
Female gender	25/58 (43.1%) (5 did not respond)
Past medical history	
Hypertension	7/63 (11,1%)
DM	1/63 (1.6%)
COPD	3/63 (4.8%)
Immune‐suppression	None (0.0%)
Missing data	5/63 (7.9%)
On regular medications	8/58 (5 did not respond)
Years of mobile ECMO experience	
<2 years	14/63 (22.2%)
>2 years	42/63 (66.7%)
Missing data	7/63 (11.1%)
Number of COVID‐19 ECMO missions involved in	
<10 missions	37/63 (58.7%)
>10 missions	18/63 (28.6%)
Missing data	8/63 (12.7%)
PPE training	57/63 (90.5%)
Unsure	2/63 (3.2%)
Missing	4/63 (6.3%)
Confirmed COVID‐19 infection	2/63 (3.2%)
Transport related injuries	None

Abbreviations: CCP, critical care paramedic; COPD, chronic obstructive pulmonary disease; DM, diabetes mellitus; ECMO, extracorporeal membrane oxygenation.

During the study period, only two of the mobile ECMO team members tested positive for COVID‐19 by the lateral flow immunoassay rapid test. In addition, none of the HCWs reported an injury or incident associated with the missions. No untoward patient transport incidents were reported to the ECMO Transport program lead (Ahmed Labib).

## 4 DISCUSSION

Our report demonstrates a low risk of COVID‐19 cross‐infection to HCWs undertaking cannulation and transporting patients with COVID‐19 supported with ECMO. Over 10 months period, only two out of 63 members of the interprofessional mobile ECMO team tested COVID‐19 positive. Transportation and retrieval on ECMO are challenging and necessitate the implementation of the interprofessional, well‐trained team, checklists, and dedicated equipment. Current guidelines also recommend a hub‐and‐spoke model to better utilize resources and centrcentralizealise this highly demanding service.^22^

The risk of transmission of COVID‐19 infection to HCWs cannot be underestimated. Globally, nearly 4% of patients reported to be infected with COVID‐19 were HCWs.^23^ Although the data was not complete, as all countries do not report HCW‐specific data, this accounted for over 150,000 COVID‐19 infected HCWs, of whom 1413 passed away. From the reported data at that early stage of the pandemic, family physicians were the most affected specialty by the disease, probably as they were often the first point of contact of patients starting to feel unwell.^23^

It is crucial to maintain staff safety and implement appropriate measures to prevent cross infection at all times and particularly during a pandemic where the number of cases is soaring, and the need for workforce across the health system is at a maximum. Transportation of COVID‐19 supported with ECMO presents the team with a multitude of practical, technical, logistical, and resource management challenges. Prior planning, effective communication, checklists, policies, training, and education are key elements to ensure staff safety and sustain the service.^24^, ^25^, ^26^, ^27^

In a recent Italian report of VV‐ECMO cannulation and interhospital transfer of 36 COVID‐19‐positive cases, none of the HCWs reported COVID‐19 infection. However, the authors did not explain how this was investigated.^28^

Another report from the USA concerning 19 road and 3 air transfers after ECMO implantation for severe COVID‐19 ARDS stated that staff involved tested negative during routine screening (by nasal PCR), but there was a limited description of the transport process and duration of exposure.^29^ However, much of these limitations have been addressed in another study pertaining to the transportation of only five COVID‐19 ECMO patients over a median distance of about 30 km.^27^ There appears to have been a focus on education, processes, and proper PPE compliance, and as a result, none of the ECMO team members were known to have been contaminated with COVID‐19. That report did not specify what, if any, testing measures were put in place for the ECMO team members, so some may have been contaminated and remained asymptomatic. The aforementioned reports^28^, ^29^ are in accordance with a multicentric study that suggested mobile ECMO does not add additional hazard to the ECMO transport team.^30^

Another study demonstrated a very low risk of COVID‐19 contamination to EMS and reported an incidence of 0.57 infections/10,000 person‐days over a similar study period.^31^

Our study, however, is based on a higher number of COVID‐19 ECMO patients transport missions and provides a more detailed description of the transport process, time of exposure, and examination of possible risk factors for HCWs cross‐infection. We report over a period during which vaccines were not yet available in the State of Qatar to protect the general population and HCWs, and hence should make everyone more cautious. Only two of the HCWs involved in our mobile COVID‐19 ECMO program tested positive for the virus during the study period. No HCW injury or incident associated with the missions was reported. This is despite a high number of missions and prolonged and repeated encounters with COVID‐19 patients.

## 5 LIMITATIONS

Our study has several limitations; it is a single‐center, retrospective observational study and subject to all biases associated with such design. Second, the number of cases and subjects is small, which makes the generalisability of the findings less likely. Akin to other studies, it was not possible to determine if those infections occurred during the transport missions, during other interactions with COVID‐19 patients in the ICU, or outside of the work context due to the prevalence of COVID‐19 within the community at the time. Ultimately, we hope that our retrospective cohort study can be complemented by further similar studies from other ECMO centers to help identify best practices and formulate further recommendations in relation to handling COVID‐19 patients in an ECMO transport context.

## 6 CONCLUSION

Transport of COVID‐19 patients on ECMO is challenging and requires meticulous attention to detail to ensure the safety of the patient and the whole team involved in the process. Aspects of cannulation and transportation involve potentially aerosol‐generating procedures that are undertaken under stressful conditions of hypoxia and clinical instability, which may add to the risk of virus transmission. Therefore, strict measures of PPE usage should be observed before, during, and after patient transportation to maintain staff and public safety. The safety measures make the process more complex than with other ECMO patients and could increase the duration of the missions but they are crucial. Our study demonstrates that COVID‐19 patients can be safely transported. Training and strict adherence to the safety measures presented above ensured that of the 63 different healthcare professionals involved in the transport and retrieval of the 34 COVID‐19 ECMO patients, only two eventually tested positive to COVID‐19, although we cannot determine for sure when and how they contracted COVID‐19. This satisfactory outcome was achieved while keeping these acutely ill patients safe from any untoward event.

## AUTHOR CONTRIBUTIONS

**Rabee Tawel**: Data curation; investigation; methodology; writing – original draft; writing – review and editing. **Lubna Altawil**: Data curation; formal analysis; validation; writing – original draft; writing – review and editing. **Sunil Hassan Koya**: Writing – original draft; writing – review and editing. **Hani Jaouni**: Writing – original draft; writing – review and editing. **Guillaume Alinier**: Investigation; methodology; validation; writing – original draft; writing – review and editing. **Abdulqadir J. Nashwan**: Investigation; methodology; writing – original draft; writing – review and editing. **Ahmed Labib**: Conceptualization; data curation; formal analysis; methodology; supervision; writing – original draft; writing – review and editing.

## CONFLICTS OF INTEREST

Abdulqadir J. Nashwan is an Editorial Board member of Health Science Reports and coauthor of this article. He is excluded from editorial decision‐making related to the acceptance of this article for publication in the journal. The authors declare no conflict of interest.

## ETHICS STATEMENT

The study was approved by the Medical Research Center (MRC)—Institutional Review Board (IRB) at Hamad Medical Corporation (MRC‐01‐21‐029). The study has been conducted in accordance with the ethical standards noted in the 1964 Declaration of Helsinki and its later amendments or comparable ethical standards.

## TRANSPARENCY STATEMENT

The lead author Abdulqadir J. Nashwan affirms that this manuscript is an honest, accurate, and transparent account of the study being reported; that no important aspects of the study have been omitted; and that any discrepancies from the study as planned (and, if relevant, registered) have been explained.

## Supporting information

Supplementary information.

## Data Availability

All data generated during this study are included in this published article.
