# Supplementary material for: Risk of COVID‐19 infection among mobile extracorporeal membrane oxygenation team
Source: Health Sci Rep. 2022 Dec 8;6(1):e981. doi: 10.1002/hsr2.981 (PMC9731299; doi:10.1002/hsr2.981)
Supplement: Supplementary file 1 — Supplementary information. [file HSR2-6-0-s001.docx]

**Supplementary Electronic Material**

| - Institutional protocols must be in place for the management of COVID-19 patients - All ECMO transport and retrieval team members should be mask fitted for N95 respirator/filtering face piece 2 (FFP2), powered air purifying respirators (PARR) or equivalent - ECMO Transport and retrieval team members must don full Personal Protective Equipment (PPE) prior to patient encounter (Figure 1) - Specialized EMS vehicle with negative pressure venting must be used for the mission - Team self-sufficient in all PPE equipment for the duration of the mission with redundancy in PPE supply - The team should review the information about the patient for which the mission has been arranged (identification, current condition, co-morbidities, location, receiving facility readiness…) prior to departure. |
| --- |

Panel1: suggested ECMO COVID-19 patient pre-transport precautionary measures

| - The cannulation should preferably be performed in a designated COVID-19 area, if available, but this has to be balanced against the risk of moving the patient within the facility. - The most experienced clinicians available should perform the cannulation. - Avoid overcrowding in the cannulation area (Figure 2). - The cannulation team must don full airborne, standard, and contact PPE (Figure 1). - The team must be fully prepared with all equipment, medications, and ancillaries before entering the cannulation area. It may be prudent to attach an automated external chest compression device to the patient before commencing cannulation. - A dedicated person in full PPE should be on stand-by outside the cannulation room to support the team with any additional equipment or medications they may request. |
| --- |

Panel 2: Recommendation for the safe cannulation of COVID-19 patients

| - A checklist must be used prior to patient transport with particular emphasis on PPE. - Clearance of transport route by security personnel and immediate availability of destination for example CT scanner. - High efficacy particulate air (HEPA) filter must be added to the endotracheal tube (ETT) and expiratory limb of the mechanical ventilator. - All efforts should be made to minimize disconnection of the breathing circuit. - Ensure tight fitting of all the components of the breathing circuit. - Clamp the ETT before any disconnection and/or reconnection. - Have a plan B and C before embarking on the journey and discuss with the team various potential eventualities and how the team plans to manage these. - Prepare equipment and medications in advance and in anticipation of emergencies. - Keep intra-hospital transport to the bare minimum and consider it only for essential diagnostic or therapeutic indications. Maintain all PPE precautions at all times. - Alert the central command centre and security team of expected arrival time and any unforeseen delays. - The patient room, the cannulation room, and the route used within the facility to transport the COVID-19 patient need to be cleaned by the housekeeping team wearing full PPE. |
| --- |

Panel 3: Safety recommendations during intra-hospital transportation of COVID-19 patients

| - The security team, in surgical masks, should plan the arrival of the ECMO transport and retrieval team and ensure clearance of the entire designated route to the patient room. - The ECMO transport and retrieval team members need to doff their PPE at base after completing the mission under the supervision of a trained safety officer to ensure it is done safely. - The dedicated route and elevators in the receiving facility need to be cleaned by the housekeeping team donning full PPE. - The ambulance needs to undergo deep cleaning as per local protocol. |
| --- |

Panel 4: Additional safety checks specific to the post-transportation phase of COVID-19 ECMO patients.
